# A retrospective study examining the association between polypharmacy and complications after laparoscopic surgery for colorectal cancer

**DOI:** 10.1186/s40780-024-00369-3

**Published:** 2024-08-02

**Authors:** Takashi Watanabe, Shota Kashiwagura, Ryusuke Ouchi, Kensuke Usui, Chikashi Shibata, Kouji Okada

**Affiliations:** 1 Division of Clinical Pharmaceutics and Pharmacy Practice, Tohoku Medical and Pharmaceutical University, 1-15-1, Fukumuro, Miyagino-ku, Sendai, 983-8536 Japan; 2 Department of Pharmacy, Tohoku Medical and Pharmaceutical University Hospital, Sendai, 983-8512 Japan; 3 Department of Gastroenterological Surgery, Tohoku Medical and Pharmaceutical University Hospital, Sendai, 983-8512 Japan

**Keywords:** Polypharmacy, Postoperative complications, Laparoscopic surgery, Colorectal cancer

## Abstract

**Background:**

Polypharmacy is an escalating public health concern across various healthcare settings worldwide. We aimed to comprehensively investigate postoperative complications after laparoscopic surgery for colorectal cancer and explore their association with polypharmacy. As laparoscopic surgery is widespread, clarifying the association between polypharmacy and postoperative complications is clinically important.

**Methods:**

We retrospectively surveyed the medical charts of adult inpatients who underwent laparoscopic surgery for colorectal cancer at Tohoku Medical and Pharmaceutical University Hospital between April 2019 and March 2023. Postoperative complications were determined using the Clavien–Dindo classification. We explored the factors related to postoperative complications and calculated the cut-off values for the number of medication ingredients.

**Results:**

Among the 236 patients, 32 (13.6%) developed postoperative complications. On multivariable logistic regression analysis, the number of regularly used medication ingredients (odds ratio = 1.160, 95% confidence interval 1.050–1.270, *p* = 0.002) was identified as a factor related to postoperative complications. The identified cut-off value for complications was 10 ingredients. Patients using 10 or more ingredients had approximately 3.5 times higher occurrence of postoperative complications than those using fewer than 10 ingredients (33.3% vs. 9.3%, *p* < 0.001, Fisher’s exact test).

**Conclusions:**

Our study comprehensively investigated postoperative complications and examined their association with polypharmacy. We found that the number of regularly used medication ingredients may be linked to complications following laparoscopic surgery for colorectal cancer. These findings have important implications for perioperative management and patient care, providing valuable insights that may influence clinical practices and enhance patient outcomes.

## Introduction

Polypharmacy is the concurrent use of multiple medications. A systematic review of polypharmacy definitions revealed that the term is most commonly applied when patients take five or more medications [1]. The high prevalence of multimorbidity in our aging societies often necessitates the use of multiple medications, resulting in polypharmacy. Recently, much attention has been directed toward the harmful effects of polypharmacy, including increased risk of adverse drug events, prolonged hospitalization, and increased mortality [2–6]. In Japan, the frequency of adverse drug events and falls increases when six or more drugs are used [7]. Thus, polypharmacy is an escalating public health concern across various healthcare settings worldwide. Polypharmacy is associated with the development of postoperative complications after gastrointestinal surgery, including after surgery for colorectal cancer [8].

Laparoscopic surgery is widely performed for patients with colorectal cancer and results in shorter hospital stays than open surgery [9, 10]. Despite becoming less invasive and reducing blood loss, postoperative complications, such as wound-related complications and anastomotic leakage, still occur after laparoscopic surgery [11]. Several studies have identified the risk factors for developing complications after colorectal cancer surgery [12–14]. In laparoscopic surgery for colorectal cancer, significant risk factors of postoperative complications include male gender and estimated blood loss ≥ 150 mL [15]. Furthermore, Hida et al. separately investigated risk factors for colonic and rectal cancers, suggesting that intraoperative management, such as a low operative infusion rate, is an independent risk factor for postoperative complications [16].

Souwer et al. identified polypharmacy as an important predictive factor of complications after colorectal cancer surgery [17], and Fagard et al. further reported it as an independent risk factor [18]. In contrast, Huisman et al., in a systematic review of older adult patients with cancer undergoing surgery, suggested that no association existed between polypharmacy and adverse postoperative outcomes [19]. These studies defined polypharmacy as the use of five or more drugs without delving into the details of the drugs or the degree of their effects. Additionally, these studies were not limited to laparoscopic surgery, and none has investigated the association between polypharmacy and complications after laparoscopic surgery for colorectal cancer. As laparoscopic surgery is widespread, clarifying the association between polypharmacy and postoperative complications is clinically important.

In this study, we aimed to comprehensively investigate postoperative complications after laparoscopic surgery for colorectal cancer and explore their association with polypharmacy. We considered polypharmacy to be synonymous with the use of multiple medications without specifying a specific number. This study provides information that can help prevent complications after laparoscopic surgery for colorectal cancer.

## Methods

### Study design and population

This retrospective cohort study included adults who underwent elective laparoscopic surgery for colorectal cancer at Tohoku Medical and Pharmaceutical University Hospital, a general hospital in Japan, between April 1, 2019, and March 31, 2023. The selected patients underwent laparoscopic colectomies or rectal resections for preoperatively diagnosed clinical stage III or lower colorectal cancer. Exclusion criteria included patients who 1) underwent ileostomies, colostomies, or bypass surgery or 2) were shifted to open surgery from laparoscopic surgery. Overall, 286 Japanese patients were selected in this study. We included the data of patients from admission to discharge to determine the presence or absence of postoperative complications during hospitalization. The patients were further observed for 30 days after surgery to confirm the presence or absence of readmission due to postoperative complications.

### Definitions

Postoperative complications and their types were determined based on the postoperative complication diagnostic algorithms described by Dindo et al. and Katayama et al. [20, 21]. The severity of postoperative complications was determined based on the Clavien–Dindo classification. Two patients with suspected Grade I postoperative complications (ascites and gait disturbance) improved during the follow-up observation. Since the suspected postoperative complications were not concluded by a doctor, the two patients were included in the non-occurrence group. In this survey, grade II or higher postoperative complications requiring treatment were categorized in the occurrence group based on a previous report [18]. When multiple postoperative complications occurred in a patient, the more severe one was considered. Two patients who received antibiotics because of postoperative infections (urinary tract infection and infection of unknown source with a blood culture performed) were categorized as “others” in the group with postoperative complications. We considered “wound infections” based on the Clavien–Dindo classification of surgical complications as infections occurring at surgical intervention sites (surgical site infection), following the definition by the Center for Disease Control and Prevention.

### Data collection

The following data were collected from electronic medical records for the patients who underwent laparoscopic surgery for colorectal cancer: age, sex, body weight, comorbidities (such as hypertension, diabetes mellitus, asthma, chronic obstructive pulmonary disease, collagen disease, and dementia), cancer site (colon or rectum), operative procedure (eight types), preoperative estimated glomerular filtration rate, operation time, anesthesia time, C-reactive protein level on postoperative day 1, pathological stage, and hospitalization days. Survey items related to medications were as follows: the number of regularly used oral medication ingredients (combination drugs were counted by the number of ingredients); the number of regularly used medication ingredients (including systemic patches, self-injectors, and inhalers, but excluding eye drops, nose drops, topical patches, and ointments); proportion of regularly used medicine (0–4, 5–9, 10–14, and ≥ 15 ingredients); regular use of anticoagulants (including antithrombotic drugs), benzodiazepines, corticosteroids, and immunosuppressants (no cases of taking potent opioids was observed in this survey); and administration of neoadjuvant chemotherapy. We counted the number of regularly used medication ingredients based on records of preoperative confirmation by pharmacists and included preoperatively discontinued medications such as anticoagulants. Prescription medications used continuously for > 30 days prior to hospitalization were considered regularly used medications. However, over-the-counter medications and supplements used before hospitalization and new medications used after surgery, such as anti-inflammatory analgesics, were not counted as regularly used medications.

### Statistical analysis

Univariate analysis was performed for each observation item between the groups with and without complications after laparoscopic surgery. The Mann–Whitney *U* test was conducted for discrete and continuous variables, and Fisher’s exact or Pearson’s chi-squared test for categorical variables. A multivariable analysis (logistic regression analysis) was performed for items suspected to be related to postoperative complications. We also generated a receiver operating characteristic curve to calculate the cut-off value for the number of regularly used medication ingredients associated with postoperative complications. The value with the highest total of sensitivity and specificity was set as the cut-off value. We calculated the area under the receiver operating characteristic curve as a quantitative measure of the discrimination power for postoperative complications. Fisher’s exact test was performed to compare postoperative complication incidence using the cut-off values for the number of regularly used oral medication ingredients as boundaries. Statistical significance was set at* p*-values < 0.05.

### Ethics approval

The ethics committee of the Tohoku Medical and Pharmaceutical University Hospital approved the study (no. 2022–2-027). We posted information about this study on the hospital website and gave participants the opportunity to opt-out; those who did not opt-out were considered to have provided tacit consent for study participation. This work has been carried out in accordance with The Code of Ethics of the World Medical Association (Declaration of Helsinki) for experiments involving humans.

## Results

### Study patients and postoperative complications

**Fig. 1:**
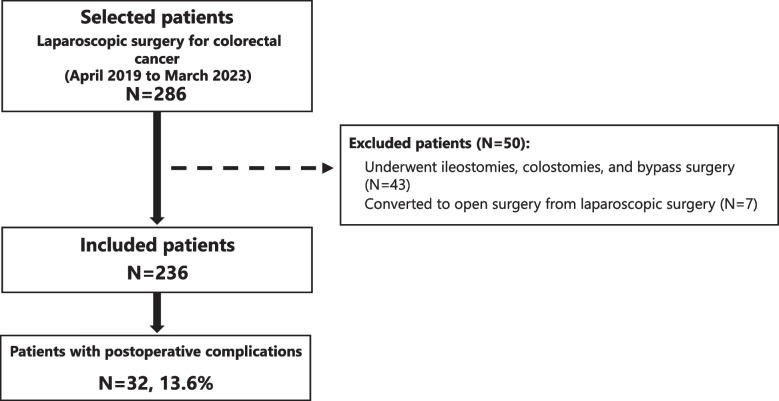
Flow chart of patient selection. *N*, number

Figure 1 shows a flowchart illustrating the patient selection process. Overall, 286 patients underwent laparoscopic surgery for colorectal cancer. The excluded cases were: 1) 43 patients who underwent ileostomies, colostomies, and bypass surgeries; 2) seven patients who converted to open surgery from laparoscopic surgery. Among the remaining 236 patients included in this study, 32 (13.6%) developed postoperative complications. The median time until the occurrence of postoperative complications was 6 (interquartile range [IQR], 3–8) days. Table 1 presents the types and severity of the postoperative complications. The most common complication was gastrointestinal anastomotic leak in eight cases (3.4%), followed by surgical site infection in five cases (2.1%) and paralytic ileus in four cases (1.7%). All gastrointestinal anastomotic leaks were grade IIIa or higher, and other postoperative complications were grade II. During the 30-day observation period after surgery, no patients were readmitted due to new postoperative complications.

**Table 1:** Classification and number of postoperative complications observed in this study

Postoperative complications	*n*	Grade II	Grade IIIa	Grade IIIb	Grade IVa
Gastrointestinal anastomotic leak	**8**		2	5	1
Surgical site infection	**5**	5			
Paralytic ileus	**4**	4			
Intestinal obstruction	**3**	3			
Pneumonia	**3**	3			
Atelectasis	**2**	2			
Dysphagia	**1**	1			
Diarrhea	**1**	1			
Chylous ascites	**1**	1			
Urinary retention	**1**	1			
Gastrointestinal anastomotic stenosis	**1**	1			
Others	**2**	2			
Total	**32**	24	2	5	1

Others: two patients received antibiotics for postoperative infections (urinary tract infection and infection of an unknown origin with blood culture performed)

*n* number

### Patient characteristics in the two groups

Table 2 summarizes the clinical data of the patients. The median age of the patients in this study was 73 ([IQR], 66–80) years, and 131 patients (55.5%) were male. The group with postoperative complications had a significantly higher median number of regularly used oral medicines and regularly used medicines (8 [IQR, 5–10] and 8 [IQR, 5–12] ingredients, respectively) than the group with no postoperative complications (5 [IQR, 3–7] and 5 [IQR, 3–8] ingredients, respectively) (*p* < 0.001 and *p* = 0.001, respectively). In addition, a significant difference was observed in the proportion of regularly used medicines. In particular, the proportion of patients using 10–14 and ≥ 15 regularly used medicines was higher in the group with postoperative complications (37.5% and 6.2%, respectively) than in the group with no postoperative complications (10.8% and 2.9%, respectively) (*p* < 0.001). Regarding the investigation of specific drugs, such as anticoagulants and benzodiazepines, no statistically significant difference was observed. The group with postoperative complications (21 [IQR, 19–29] days) exhibited significantly longer median hospitalization durations than the group without postoperative complications (13 [IQR, 12–15] days) (*p* < 0.001). The two groups did not significantly differ in other items.

**Table 2:** Patient characteristics

	Group with	Group with	*p-*value
	postoperative complications	no postoperative complications	
	(*N* = 32)	(*N* = 204)	
Age (years)	75 [69, 85]	73 [66, 79]	0.093^a)^
Male sex, *N* (%)	19 (59.4)	112 (54.9)	0.704^b)^
Body weight (kg)	61.6 [48.2, 67.9]	58.0 [49.4, 69.2]	0.812^a)^
Comorbidity			
Hypertension, *N* (%)	22 (68.8)	134 (65.7)	0.889^b)^
Heart failure, *N* (%)	11 (34.4)	48 (23.5)	0.272^b)^
Diabetes mellitus, *N* (%)	10 (31.3)	54 (26.5)	0.725^b)^
Asthma or COPD, *N* (%)	8 (25.0)	29 (14.2)	0.194^b)^
Collagen disease, *N* (%)	2 (6.3)	7 (3.4)	0.351^c)^
Dementia, *N* (%)	1 (3.1)	3 (1.5)	0.444^c)^
Cancer site			0.344^c)^
Colon, *N* (%)	28 (87.5)	161 (78.9)	
Rectum, *N* (%)	4 (12.5)	43 (21.1)	
Operative procedure			0.806^c)^
Ileocecectomy, *N* (%)	6 (18.8)	28 (13.7)	
Right hemicolectomy, *N* (%)	11 (34.4)	55 (27.0)	
Transverse colectomy, *N* (%)	1 (3.1)	4 (2.0)	
Left hemicolectomy, *N* (%)	3 (9.4)	16 (7.8)	
Sigmoidectomy, *N* (%)	7 (21.9)	58 (28.4)	
Subtotal colectomy, *N* (%)	0 (0.0)	1 (0.5)	
High anterior resection, *N* (%)	1 (3.1)	17 (8.3)	
Low anterior resection, *N* (%)	3 (9.4)	25 (12.3)	
Preoperative eGFR (mL/mL/1.73 m^2^)	62.5 [47.9, 74.5]	64.0 [52.0, 74.4]	0.621^a)^
Operation time (min)	238 [193, 268]	220 [181, 261]	0.411^a)^
Anesthesia time (min)	299 [250, 322]	274 [234, 314]	0.298^a)^
CRP at postoperative day 1 (mg/dL)	5.01 [3.73, 6.42]	4.36 [2.78, 6.09]	0.207^a)^
Pathological Stage			0.189^c)^
Stage 1, *N* (%)	9 (28.1)	93 (45.6)	
Stage 2, *N* (%)	8 (25.0)	49 (24.0)	
Stage 3, *N* (%)	15 (46.9)	61 (29.9)	
Stage 4, *N* (%)	0 (0.0)	1 (0.5)	
Regularly used oral medicine (number of ingredients)	8 [5, 10]	5 [3, 7]	< 0.001^a)^
Regularly used medicine (number of ingredients)	8 [5, 12]	5 [3, 8]	0.001^a)^
Proportion of regularly used medicine			< 0.001^c)^
0–4 ingredients (%)	7 (21.9)	92 (45.1)	
5–9 ingredients (%)	11 (34.4)	84 (41.2)	
10–14 ingredients (%)	12 (37.5)	22 (10.8)	
≥ 15 ingredients (%)	2 (6.2)	6 (2.9)	
Regular use of			
anticoagulants, *N* (%)	14 (43.8)	57 (27.9)	0.108 b)
benzodiazepines, *N* (%)	6 (18.8)	25 (12.3)	0.465 b)
steroids, *N* (%)	2 (6.3)	7 (3.4)	0.351 c)
immunosuppressants, *N* (%)	1 (3.1)	2 (1.0)	0.355 c)
Neoadjuvant chemotherapy, *N* (%)	0 (0.0)	2 (1.0)	1.000 c)
Hospitalization days (day)	21 [19, 29]	13 [12, 15]	< 0.001 a)

Discrete and continuous data are expressed as medians (interquartile ranges). Categorical data are expressed as numbers (%)

*COPD* chronic obstructive pulmonary disease, *CRP* C-reactive protein, *eGFR* estimated glomerular filtration rate, *N* number

^a)^Mann–Whitney *U* test

^b)^Pearson chi-squared test

^c)^Fisher’s exact test

### Examination of factors related to postoperative complications

Along with performing multivariable analysis, we selected age, male sex, and polypharmacy-related items for use in the logistic regression analysis. Only the “number of regularly used medication ingredients” was included in the polypharmacy-related items to address multicollinearity (Table 3). Among these, the number of regularly used medication ingredients was identified as a factor related to postoperative complications (odds ratio = 1.160, 95% confidence interval, 1.050–1.270; *p* = 0.002).

**Table 3:** Multivariable logistic regression analysis of factors related to postoperative complications

	Odds ratio	95% confidence interval	*p-*value ^a)^
Regularly used medicine (number of ingredients)	1.160	1.050–1.270	0.002
Male sex	0.876	0.397–1.930	0.743
Age	1.020	0.976–1.070	0.373

Among the 236 patients, 32 had postoperative complications and 204 did not have postoperative complications

The variance inflation factors for these items are 1.060, 1.027, and 1.080, respectively

^a)^Logistic regression analysis

### The association between polypharmacy and postoperative complications

**Fig. 2:**
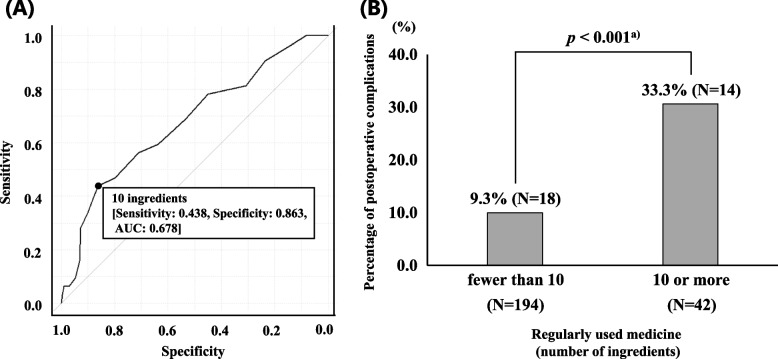
Receiver operating characteristic curve of the association between the number of regularly used medication ingredients and postoperative complications (**A**) and comparison of the postoperative complication incidence using the cut-off value as the limit (**B**). *N*, number. a) Fisher’s exact test

Drawing a receiver operating characteristic curve using the number of regularly used medication ingredients as a predictor of postoperative complications resulted in a cut-off value of 10 ingredients (sensitivity, 0.438; specificity, 0.863; area under the curve, 0.678) (Fig. 2-A). Furthermore, using this cut-off value, 9.3% (18/194) of postoperative complications occurred in the group with < 10 ingredients and 33.3% (14/42) in the group with ≥ 10 ingredients. The group receiving ≥ 10 ingredients exhibited significantly higher incidence of postoperative complications (*p* < 0.001) (Fig. 2-B).

## Discussion

We conducted a retrospective medical chart survey to examine the association between polypharmacy and complications after laparoscopic surgery for colorectal cancer. We observed that the number of regularly used medication ingredients was related to postoperative complications, with a higher incidence of these complications when using 10 or more ingredients. Polypharmacy is associated with many challenges, including an increased risk of adverse drug events. In Japan, "Guidelines for the proper use of medicines for the elderly (Ministry of Health, Labor and Welfare, May 2018)" and "Guidelines for medical treatment and its safety in the elderly (The Japan Federation of Gerontological Societies, December 2015)" have been published, emphasizing the importance of measures against polypharmacy.

Among patients who underwent laparoscopic surgery for colorectal cancer at our hospital between April 2019 and March 2023, 32 of the 236 patients surveyed experienced postoperative complications, resulting in an incidence proportion of 13.6%. This percentage was similar to that reported in a previous study that included 333 patients [22]. In previously reported randomized controlled trials, the median incidence of grade II or higher laparoscopic postoperative complications, similar to the definition in this study, was 8.8% (range 4.7–24.0%) [11]. The results in this study were within this range; however, differences in patient backgrounds and hospital characteristics accounted for the variation between reports. Among the types of postoperative complications, the frequency of gastrointestinal anastomotic leaks, the most common complication (3.4%), was similar to or lower than those previously reported [23]. These differences in the frequency of occurrence vary depending on the surgery type and cancer site. All gastrointestinal anastomotic leaks in our study were grade III or higher, and image-guided drain placement and re-anastomosis were performed under general anesthesia. Surgical site infections, the second most common complication (2.1%), were relieved with antibiotic administration. The frequency of surgical site infection after laparoscopic surgery for colorectal cancer is 12.6% [24]. However, this percentage cannot be directly compared with that in our study because it also includes anastomotic leaks and other surgical infections, such as perineal wound infections. Risk factors for each of these postoperative complications have been reported [23, 25–27]. In this study, we comprehensively investigated and analyzed postoperative complications; we will not discuss each postoperative complication.

We investigated the factors related to postoperative complications based on the hypothesis that polypharmacy is associated with the outcomes after laparoscopic surgery for colorectal cancer. Rather than simply defining polypharmacy as the administration of five or more drugs, we treated polypharmacy as synonymous with the use of multiple medications. This approach made it possible to investigate the specific degree of involvement of the number of drugs. We counted the number of regularly used oral medication ingredients and regularly used medication ingredients, including parenteral drugs. In the univariate analysis, the group with postoperative complications had a significantly higher number of both items. The two groups did not significantly differ in terms of items other than the number of medication ingredients, proportion of regularly used medicine, and hospitalization days. The finding that the group with postoperative complications had significantly longer hospitalization days was consistent with those of previous reports [15, 28]. Based on the results, we conducted a multivariable analysis using the following three items: an item suspected to be related to postoperative complications (number of regularly used medication ingredients), an item reported as a risk factor in previous studies (male sex) [15, 22], and an item reported to be associated with polypharmacy progression (age) [29, 30]. Consequently, the number of regularly used medication ingredients was identified as a factor related to postoperative complications. The greater the number of medication ingredients taken, the higher the risk of adverse drug events due to various functional changes, such as gastrointestinal peristalsis, intestinal equilibrium, and blood hemodynamics. The persistent harmful effects of multiple medications may lead to postoperative complications in patients with colorectal cancer. Alternatively, some regularly used medications may be discontinued during the perioperative period. Temporary discontinuation of these medications may lead to unstable drug efficacy, resulting in the occurrence of adverse events. The analysis was conducted using regularly used medications, including parenteral drugs, and similar results were obtained for regularly used oral medications in multivariable analysis (Data not shown). Certainly, it is necessary to consider the possibility that postoperative complications may be caused by the influence of multiple diseases (multimorbidity) that lead to polypharmacy. The present results may serve as an indicator of the occurrence of postoperative complications.

In the present study, male sex had no significant association with the incidence of postoperative complications, contrary to a previous study by Xia et al., which identified male gender and estimated blood loss ≥ 150 mL as possible risk factors for postoperative complications [15]. However, Xia et al. did not analyze polypharmacy-related items [15] and the present study did not investigate blood loss. These discrepancies may be due to differences in observation items, sample size, and characteristics of each facility. Other studies have also reported additional risk factors, such as respiratory function and sarcopenia, but these items were not evaluated in the present study [12–14]. Of note, many of the previous studies were not limited to laparoscopic surgery, making comparison of results difficult.

We calculated the cut-off value for the number of regularly used medication ingredients for postoperative complications by plotting a receiver operating characteristic curve to further examine the association of polypharmacy with postoperative complications. The cut-off value was set at 10 ingredients, the point with the highest combined sensitivity and specificity. The area under the curve was 0.678, indicating a “slightly low” predictive ability. The sensitivity and specificity for predicting postoperative complications were 44% and 86%, respectively. With a focus on specificity, the calculated cut-off value results in fewer false positives, enabling highly reliable screening for postoperative complications in patients with polypharmacy. However, our results showed high specificity but a low positive predictive value (33%). Therefore, it is challenging to discuss the clinical usefulness considering solely the calculated cut-off value, and further investigation is needed to determine an appropriate cut-off value. In our study, the group receiving 10 or more ingredients had a significantly higher incidence of postoperative complications than the group receiving fewer than 10 ingredients (approximately 3.5 times higher). This result suggests that the calculated cut-off value may be one indicator related to postoperative complications. Previous studies have defined polypharmacy as five or more regularly used medicines and compared postoperative complications by classifying them into two categories: with and without polypharmacy [1]. Therefore, directly comparing the present results with those of previous studies is impossible. Kojima et al. reported that adverse drug events significantly increased in hospitalized older adult patients taking six or more medications [7]. This study targeted older adult patients, suggesting that a decline in patients’ daily life activities and physiological functions may have had a greater effect on adverse drug events. Our study did not limit the target patients to older adults, and the 10 ingredients were calculated, considering the influence of medicines on all age groups. The details of the association with polypharmacy, including specific drugs, on postoperative complications, need to be further investigated in the future. The results of this study suggest that thorough perioperative management and close attention to the postoperative course may prevent or ensure early detection of postoperative complications in patients with polypharmacy.

This study had some limitations. First, this was a single-hospital retrospective observational study, and potential selection bias could not be avoided. Second, this study did not investigate the general conditions of the patients or the epidural anesthesia effects. These survey items may influence postoperative complication occurrence. Additionally, we did not investigate items such as blood loss and respiratory function or sarcopenia [12–15]. Studies using these parameters were not limited to laparoscopic surgery; however, they may influence postoperative complication occurrences. Third, we did not evaluate the association between multimorbidity and the occurrence of postoperative complications. In other words, the influence of multimorbidity that leads to polypharmacy cannot be excluded. Correction for the effect of multimorbidity was necessary; the Charlson Comorbidity Index and others should be utilized to discuss the polypharmacy in the results after adjusting for multimorbidity. In this study, it was difficult to calculate accurate values of the Charlson Comorbidity Index for many patients. Future research should adjust for multimorbidity in evaluating the influence of polypharmacy on postoperative complications. By carefully considering appropriate pharmacotherapy for comorbidities and keeping in mind the correction of polypharmacy, safe and effective medical care can be provided to patients.

## Conclusions

Our study comprehensively investigated postoperative complications and examined their association with polypharmacy. We found that the number of regularly used medication ingredients may be related to complications after laparoscopic surgery for colorectal cancer. In patients with polypharmacy, thorough perioperative management and close monitoring of the postoperative course may help prevent or ensure early detection of complications. This study provides valuable insights that may influence clinical practices and enhance patient outcomes.

## Data Availability

The data cannot be shared according to regulations given by the ethics committee of the Tohoku Medical and Pharmaceutical University Hospital.

## Abbreviation

IQR: Interquartile range
